# Patterns of chromosomal variation in Mexican species of *Aeschynomene* (Fabaceae, Papilionoideae) and their evolutionary and taxonomic implications

**DOI:** 10.3897/CompCytogen.v14i1.47264

**Published:** 2020-03-11

**Authors:** Fernando Tapia-Pastrana, Alfonso Delgado-Salinas, Javier Caballero

**Affiliations:** 1 Facultad de Estudios Superiores Zaragoza, Universidad Nacional Autónoma de México, Laboratorio de Genecología, Batalla 5 de Mayo s/n esquina Fuerte de Loreto, Col. Ejército de Oriente, Iztapalapa, C.P. 09230, Ciudad de México, Mexico Universidad Nacional Autónoma de México México Mexico; 2 Instituto de Biología, Departamento de Botánica, Universidad Nacional Autónoma de México, Apartado Postal 70-233, 04510, Cd. de México, Mexico Universidad Nacional Autónoma de México México Mexico; 3 Jardín Botánico, Instituto de Biología, Universidad Nacional Autónoma de México, Circuito Campos Deportivos, Ciudad Universitaria, Coyoacán 04510, Cd. de México, Mexico Universidad Nacional Autónoma de México México Mexico

**Keywords:** infraspecific taxa, karyotypes, Leguminosae, New World, satellites, secondary constrictions

## Abstract

A cytogenetic analysis of sixteen taxa of the genus *Aeschynomene* Linnaeus, 1753, which includes species belonging to both subgenera *Aeschynomene* (Léonard, 1954) and *Ochopodium* (Vogel, 1838) J. Léonard, 1954, was performed. All studied species had the same chromosome number (2n = 20) but exhibited karyotype diversity originating in different combinations of metacentric, submetacentric and subtelocentric chromosomes, chromosome size and number of SAT chromosomes. The plasticity of the genomes included the observation in a taxon belonging to the subgenus Aeschynomene of an isolated spherical structure similar in appearance to the extra chromosomal circular DNA observed in other plant genera. By superimposing the karyotypes in a recent phylogenetic tree, a correspondence between morphology, phylogeny and cytogenetic characteristics of the taxa included in the subgenus Aeschynomene is observed. Unlike subgenus Aeschynomene, the species of *Ochopodium* exhibit notable karyotype heterogeneity. However the limited cytogenetic information recorded prevents us from supporting the proposal of their taxonomic separation and raise it to the genus category. It is shown that karyotype information is useful in the taxonomic delimitation of *Aeschynomene* and that the diversity in the diploid level preceded the hybridization/polyploidization demonstrated in the genus. The systematic implications of our results and their value can be extended to other Dalbergieae genera as knowledge about the chromosomal structure and its evolution increases.

## Introduction

*Aeschynomene* Linnaeus, 1753 (Fabaceae, tribe Dalbergieae s. l. Cardoso et al. 2013) is a diverse genus of subfamily Papilionoideae (Papilionoid legumes) distributed in the tropics and subtropics of the world (Lavin et al. 2001, Klitgaard and Lavin 2005). The number of new described species has increased rapidly in last decades (Queiroz and Cardoso 2008, Delgado-Salinas and Sotuyo 2012, Silva and Antunes 2014, Antunes and Silva 2017, Chaintreuil et al. 2018) and currently 170 scientific names are accepted according to The Plant List (http://www.theplantlist.org/tpl/search?q=Aeschynomene). The genus *Aeschynomene* has evolved in different ecological niches and includes herbaceous forms, annual and perennial shrubs as well as trees up to 8 meters high, with compound pinnate leaves and papilionoid flowers that are generally self-pollinated, although there is cross-pollination by bees (Arrighi et al. 2014, Carleial et al. 2015). Half of the species are found in the New World, the proposed center of origin of the genus (Chaintreuil et al. 2013, 2018), mainly in Mexico and South America (Rudd 1955); the other half is found in the tropical regions of Africa (center of secondary diversification), SE Asia, Australia and the Pacific Islands (Arrighi et al. 2013, Chaintreuil et al. 2013, LPWG 2013). The current infrageneric classification of New World *Aeschynomene* largely follows the taxonomic groups proposed by Rudd (1955, 1981) who recognized 67 taxa, although recent estimates suggest the existence of 86 species (Fernandes 1996, Klitgaard and Lavin 2005). Subgenus Aeschynomene Léonard, 1954 includes hydrophytes herbs and shrubs with peltate stipules, fruits with articles separated by septa, and bilabiate calyx, growing in swamps, wet meadows, river channels and streams. Species of subgenus Ochopodium (Vogel, 1838) J. Léonard, 1954 are terrestrial herbs, shrubs and trees with basifixed stipules, fruits with articles separated by an isthmus, and campanulate calyx with five subequal teeth, occur in savannahs, pine and oak groves, rocky slopes, sandy beaches and dry places (Rudd 1955, Fernandes 1996).

In Mexico grow 31 species and infraspecific taxa (including several endemisms) distributed in both Atlantic and Pacific slopes as well as in the center of the country. Those corresponding to subgenus Aeschynomene are included in three of five series that make up the group (Americanae- plants with flexible edaphic requirements; Sensitivae and Indicae- predominantly hydrophytic). Those corresponding to subgenus Ochopodium are included in three of the four series (Pleuronerviae, Scopariae and Viscidulae) and occupy mesic and subxeric habitats (Rudd 1955, Fernandes 1996).

Traditionally *Aeschynomene* was included in the tribe Aeschynomeneae, however molecular evidence place it in the most widely circumscribed tribe Dalbergieae*sensu lato* (Lavin et al. 2001, Wojciechowski et al. 2004), and show that it is a paraphyletic group with species that are nested separately in two well supported clades (Lavin et al. 2001, Ribeiro et al. 2007, Cardoso et al. 2012). These studies suggest that *Ochopodium* should be raised to the category of genus as a sister group of *Machaerium* Persoon, 1807. Morphological studies on floral ontogeny also support this proposal (Sampaio et al. 2013). Additionally, Chaintreuil et al. (2013) showed that the aquatic and semiaquatic species of *Aeschynomene* (series Indicae and Sensitivae) form the monophyletic clade Nod-independent, whose taxa are nodulated on roots and stems by photosynthetic Bradyrhizobium strains lacking the nod ABC genes necessary for the synthesis of Nod factors (Giraud et al., 2007), while *A. americana* Linnaeus, 1753 and *A. villosa* Poiret, 1816 (series Americanae) are sisters within American clade Nod-dependent, whose hydrophytes do not nodulate on stems. However, the number of species sampled in previous studies is still limited, and the inclusion of African species and related genera indicates that subgenus Aeschynomene is also paraphyletic. In addition to this generic delimitation problem, there is a need to understand the biology and taxonomy of several polymorphic species (e. g., *Aeschynomene americana* and *A. villosa*), which justifies a more comprehensive taxonomic revision of the genus (LPWG 2013).

The cytogenetics studies of the genus showed that there is agreement on the basic number x = 10 (Bir and Kumari 1977, Coleman and Demenezes 1980, Bairiganjan and Patnaik 1989) and 2n = 20 for most species (Renard et al. 1983, Vanni 1983, Kumari and Bir 1990, Seijo and Vanni 1999). Arrighi et al. (2014) used molecular, cytogenetic methods and measure of nuclear DNA content, to analyze the role of polyploidy in *Aeschynomene* New World species of the Nod-independent clade from North America. In addition to providing new records of chromosome numbers, they revealed multiple hybridization/polyploidization events, highlighting the prominent role of allopolyploidy in the diversification of *Aeschynomene* Nod-independents. Chaintreuil et al. (2016a) studied African *Aeschynomene* species and their data support the idea that the whole African group is fundamentally tetraploid (4x) with a common AB genome structure, indicating that a single ancient polyploid event occurred that preceded its diversification. They also revealed the allopolyploid origin of *A. afraspera* J. Léonard, 1954 (2n = 8x = 76) and *A. schimperi* Hochstetter ex A. Richard, 1847 (2n = 8x = 56), where variations in the number of chromosomes also indicated possible dysploidy/aneuploidy events. Therefore, it is necessary to expand the sampling of some taxa or clades of *Aeschynomene* to delimit morphologically similar taxa that show geographically based intraspecific genetic diversity or that exhibit cytotypes (Brottier et al. 2018, Chaintreuil et al. 2018).

Although polyploidy and dysploidy play an important role in the evolution of genomes, chromosomal rearrangements also participate in the evolution of genome size and in the remodeling of its architecture, thus contributing to the diversification of genomes (Rieseberg 2001, Raskina et al. 2008, Faria and Navarro 2010, Chaintreuil et al. 2016a). In this sense the karyotypic analysis in *Aeschynomene* has been little explored, which makes it impossible to know the magnitude and direction of the karyological evolution, the mechanisms involved in the diversification of the genomes and their systematic and phylogenetic implications. This encouraged us to perform a cytogenetic analysis of selected species and infraspecific Mexican taxa of *Aeschynomene*, together with *A. rudis* Bentham, 1843 from Argentina to investigate (1) its chromosomal and karyotype diversity (2) its relation to the current taxonomic classification and molecular phylogeny; (3) evaluate interspecific delimitations and infraspecific differences, particularly in taxa with taxonomic difficulties and (4) compare the cytogenetic information with the recent morphological and molecular evidence to improve the taxonomy and offer an opinion on the separation of *Ochopodium* as a genus.

## Material and methods

### Plant material

Together 17 accessions including ten species and four varieties of the genus *Aeschynomene*, as well as two populations that could potentially represent new species or varieties herein categorized as Aeschynomene sp. prope americana and Aeschynomene sp. prope villosa were examined in this study (Table 1). The vouchers of the studied specimens were deposited in the National Herbarium (MEXU) of the Instituto de Biología, UNAM, and in the Herbarium of the Facultad de Ciencias Naturales (MCNS), Universidad Nacional de Salta Argentina.

**Table 1.** Geographical data on studied *Aeschynomene* accessions.

Species	Original location	Habitat	Latitude / Longitude
*Aeschynomene americana* Linnaeus, 1753	MEX, Jalisco, Municipio de la Huerta	Semiaquatic	19.4833333, -105.016667
A. americana var. flabellata Rudd, 1955	MEX, Guerrero, Municipio de Chilapa de Álvarez	Semiaquatic	17.9833333, -99.0333333
A. americana var. glandulosa (Poiret) Rudd, 1955	MEX, Guerrero, Municipio de Cocula	Semiaquatic	18.2333333, -99.15
Aeschynomene sp. prope americana	MEX, Oaxaca, Municipio de Santiago Pinotepa Nacional	Semiaquatic	16.35, -98.05
*A. amorphoides* Rose, 1894	MEX, Jalisco, Municipio de la Huerta	Terrestrial	19.4833333, -105.016667
*A. ciliata* Vogel, 1838	MEX, Veracruz, Municipio de Catemaco	Semiaquatic	18.4166667, -95.1
*A. deamii* Robinson et Bartlett, 1909	MEX, Tabasco, Municipio de Jonuta	Semiaquatic	18.0833333, -92.1333333
*A. evenia* C.Wright, 1869	MEX, Guerrero, Municipio de Coyuca de Catalán	Semiaquatic	18.3166667, -100.7
*A. lyonnetii* Rudd, 1989	MEX, Guerrero, Municipio de Tepecoacuilco de Trujano	Terrestrial	18.3, -99.15
*A. paniculata* Willdenow ex Vogel, 1838	MEX, Guerrero, Municipio de Chilpancingo de los Bravo	Terrestrial	17.55, -99
*A. rudis* Bentham, 1843	ARG, Provincia de Salta	Semiaquatic	-23.15, -64.05
*A. scabra* G.Don, 1832	MEX, Guerrero, Municipio de Arcelia	Semiaquatic	18.3, -100.283333
*A. sensitiva* Swartz, 1788, I	MEX, Guerrero, Municipio de Atoyac de Álvarez	Semiaquatic	17.2, -100.416667
*A. sensitiva* Swartz, 1788, II	MEX, Veracruz, Municipio de Texistepec	Semiaquatic	17.8166667, -94.15
A. villosa var. villosa Poiret, 1816	MEX, Oaxaca, Municipio de Santiago Pinotepa Nacional	Semiaquatic	16.3333333, -98.05
A. villosa var. longifolia (Micheli) Rudd, 1955	MEX, Veracruz, Municipio de Jáltipan de Morelos	Semiaquatic	17, -94
Aeschynomene sp. prope villosa	MEX, Oaxaca, Municipio de Santiago Pinotepa Nacional	Semiaquatic	16.3333333, -98.05

### Chromosome and karyotype procedures

The mitotic cells were gathered from radicular meristems of seeds that come from at least six individuals per accession, germinated in Petri dishes lined with cotton moistened in distilled water. Chromosomes at metaphase and prometaphase were obtained following the splash method by Tapia-Pastrana and Mercado-Ruaro (2001) briefly described as follows: the meristems were separated from the root when it reached between 3–5 mm in length and were pretreated with fresh solution of 0.002M 8-hydroxyquinoline for 5 h at room temperature and fixed in the fixative Farmer´s solution (ethanol : acetic acid, 3 : 1). Then they were treated in a mixture of 2% cellulase (w/w, Sigma) and 20% pectinase (v/w, Sigma) in 75 mM KCl for 2 h at 37 °C. After centrifugation at 1500 rpm for 10 min, the cell pellet was transferred to 75 mM KCl solution for 20 min at 37 °C. After two successive rinses with KCl solution they were again fixed in Farmer´s solution and subsequently rinsed twice more. One or two drops of the suspension of pellet were placed on clean slides, air dried and stained in 10% Giemsa for 10 min. Preparations were made permanent using a synthetic resin. At least ten well spread metaphase plates were photographed (AxioCam ERc5s Zeiss) from each collection, using a Carl Zeiss Axioscope A1 and analyzed for chromosome number. Five photographs of metaphases with chromosomes having comparable degrees of contraction were utilised to obtain mean values in the following chromosomal parameters: the difference in length between the longest chromosome and shortest chromosome (Range), total haploid chromosome length (THC), average chromosomal size (AC) and ratio of the longest/shortest chromosome (Ratio, L/S). The index of asymmetry (TF) was obtained following Huziwara (1962) and the centromeric index (CI) was established by the formula CI = [SA/SA + LA)] × 100. The chromosomes were classified according to Levan et al. (1964) and the classification of the satellites followed Battaglia (1955). Only the preparations of two species, *Aeschynomene evenia* C.Wright, 1869 and *A. scabra* G.Don, 1832 were recorded in digital images and analyzed in free microscope software Zen lite (Zeiss Microscopy). Remaining taxa were recorded on photographs with the same magnification and the chromosome sizes were estimated using a digital calibrator Mitutoyo Digimatic Caliper CD-6" BS. In the estimation of chromosomal sizes the satellite size was not considered. Karyotypes were prepared from photomicrographs by cutting out individual chromosomes, arranging them in descending order of length and matching on the basis of morphology.

### Data analysis and processing

To analyze the patterns of chromosomal variation in the studied taxa, grouping and sorting techniques were used through the NTSYS-PC program version 2.21 developed by Rohlf (2012). A basic data matrix was constructed with 10 chromosome characters, including the total number and number of particular types of chromosomes (m, sm and st), THC, AC, Range, Ratio, TF, and CI (Table 2) and standardized by the linear transformation method and a character correlation matrix was calculated. The variation patterns were evaluated by a principal component analysis (PCA) performed on the correlation matrix. The significance of the groupings was later proven by an analysis of discriminant functions (DFA).

**Table 2.** Matrix of cytogenetic data on taxa under study.

Subgenus Aeschynomene	2n	Karyotype formula	THC (µm)	AC (µm)	Range (µm)	Ratio (L/S)	TF	CI
*A. americana*	20	8m + 1sm + 1st	12.85	1.28	0.86	1.99	40.75	39.88
A. americana var. flabellata	20	8m + 1sm + 1st	13.92	1.39	0.77	1.74	42.02	41.40
A. americana var. glandulosa	20	8m + 1sm + 1st	15.86	1.58	0.95	1.98	43.23	42.12
Aeschynomene sp. prope americana	20	8m + 1sm + 1st	16.54	1.64	0.98	1.86	43.06	42.13
A. villosa var. villosa	20	4m + 4sm + 2st	14.16	1.41	0.98	2.16	35.17	34.07
A. villosa var. longifolia	20	4m + 6 sm	13.68	1.36	0.91	2.01	36.98	36.79
Aeschynomene sp. prope villosa	20	7m + 2sm + 1st	15.90	1.58	1.09	2.06	40.40	39.79
*A. sensitiva* I	20	9m + 1sm	16.65	1.66	0.90	1.72	42.82	43.11
*A. sensitiva* II	20	9m + 1sm	15.66	1.56	0.77	1.63	43.25	42.97
*A. deamii*	20	8m + 2sm	20.82	2.07	1.01	1.65	41.35	41.48
*A. scabra*	20	10m	15.71	1.56	0.66	1.51	45.54	45.54
*A. evenia*	20	7m + 3sm	14.15	1.41	0.82	1.82	42.04	41.50
*A. rudis*	20	8m + 2st	11.39	1.13	0.60	1.74	39.64	39.13
*A. ciliata*	20	7m + 3sm	15.71	1.56	0.90	1.82	41.13	40.83
**Subgenus Ochopodium**								
*A. paniculata*	20	3m + 7sm	19.28	1.92	1.82	2.52	36.56	36.63
*A. lyonnetii*	20	9m + 1sm	21.86	2.18	1.67	2.33	43.78	41.88
*A. amorphoides*	20	8m + 2st	22.41	2.24	1.46	1.98	39.66	37.61

## Results

### Karyotype diversity

**Figure 1.**
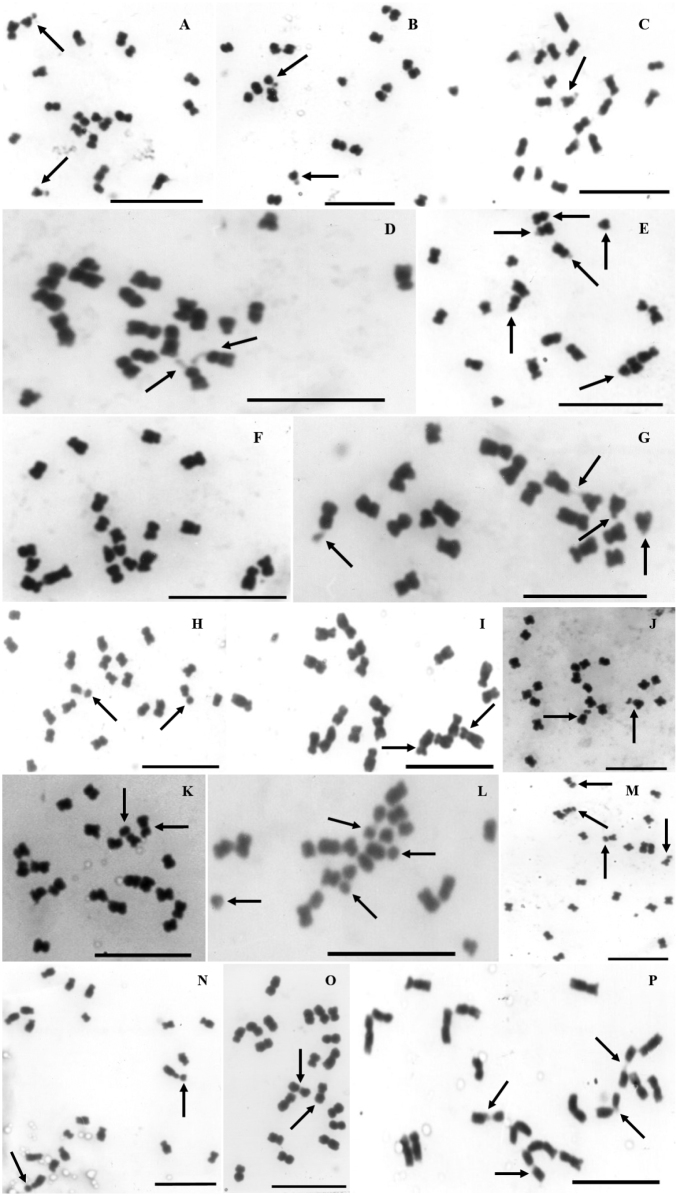
Mitotic metaphase cells of *Aeschynomene*, all the taxa with 2n = 20. Subgenus Aeschynomene**A–G** series Americanae **A***A. americana***B**A. americana var. flabellata**C**A. americana var. glandulosa**D**Aeschynomene sp. prope americana**E**A. villosa var. villosa**F**A. villosa var. longifolia**G**Aeschynomene sp. prope villosa**H, I** series Sensitivae **H***A. sensitiva***I***A. deamii***J–M** series Indicae **J***A. scabra***K***A. evenia***L***A. rudis***M***A. ciliata*Subgenus Ochopodium**N–P** series Pleuronerviae **N***A. paniculata***O, P** series Scopariae **O***A. lyonnetii***P***A. amorphoides*. The arrows point to the chromosomes with satellites. Scale bars: 10 μm.

All the taxa exhibited constancy in the chromosome number 2n = 20. Chromosome complements with metacentric (m) and submetacentric (sm) chromosomes and subtelocentric chromosomes (st, no more than two pairs per complement), predominated. Together 10 karyotypic formulae were found. The most frequent karyotype formulae were 8m + 1sm + 1st (studied taxa of series Americanae of *Aeschynomene*) and 9m + 1sm (both populations of *A. sensitiva* Swartz, 1788, and one species of the series Scopariae of *Ochopodium*) (Fig. 1, Table 2). Other taxa had its unique karyotype formula. In accordance with the above, both the CI (34.07 to 45.54) as well as TF (35.17 to 45.54) indicated slightly asymmetric karyotypes in *Aeschynomene* (Table 2). All the complements contained chromosomes with secondary constrictions in the short arms associated with microsatellites (all taxa in series Americanae; Fig. 1A–G) or with macrosatellites (series Sensitivae, Indicae, Pleuronerviae and Scopariae; Fig. 1H–P), that can be located in metacentric chromosomes (e.g., Aeschynomene sp. prope americana, A. villosa var. villosa Poiret, 1816 and Aeschynomene sp. prope villosa), submetacentric (e.g., *A. paniculata* Willdenow ex Vogel, 1838) or subtelocentric (e.g., *A. americana* and *A. rudis*), and with a maximum number of six in A. villosa var. villosa. Only *A. paniculata* (Fig. 1N) exhibited macrosatellites situated on the largest pair of chromosomes (sm). Thus SAT chromosomes varied both in number (1 to 3 pairs) and position within the karyotypes, commonly they were occurred in the smallest chromosomal pair but also in the first pair. Variation in the size of the satellites was also observed and the most notable case was *A. lyonnetii* Rudd, 1989 where their location in the smallest chromosomal pair was only achieved after clearly observing both the centromere and the secondary constriction, which prevented a misinterpretation due to its large size (Fig. 1O).

The chromosomal complements of the analyzed taxa were small sized chromosomes (Lima de Faria 1980) which can be separated into two groups based on their size: (i) complements with average chromosomal size (AC) close to 1.5 μm (e.g., series Americanae, Sensitivae and Indicae of subgenus Aeschynomene) and (ii) those with AC close to 2.0 μm (e.g., series Pleuronerviae and Scopariae of subgenus Ochopodium) (Table 2). Intriguingly *A. deamii* Robinson et Bartlett, 1909 with AC = 2.07 μm represented a notable case in the subgenus Aeschynomene.

### Chromosomal comparisons within a phylogenetic framework

**Figure 2.**
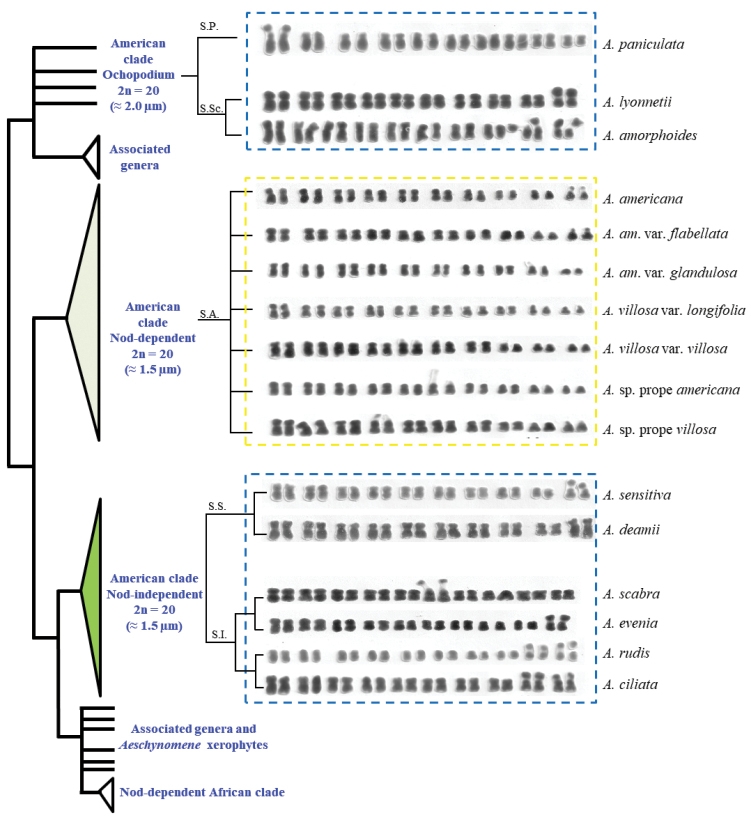
Karyotypes of the studied *Aeschynomene* taxa superimposed on a simplified and stylized phylogenetic tree (modified from Chaintreuil et al. 2016a). Abbreviations: S. P. – series Pleuronerviae; S. Sc. – series Scopariae; S. A. – series Americanae; S. S. – series Sensitivae; S. I. – series Indicae. Blue dashed lines frame the karyotypes exhibiting macrosatellites; the yellow ones, those with microsatellites.

The members of clades distinguished by Chaintreuil et al. (2016a) had similar values in several parameters. It can be seen that within subgenus Aeschynomene, series Americanae includes *Aeschynomene americana* and its varieties, as well as populations labeled as Aeschynomene sp. prope americana, A. villosa var. villosa, A. villosa var. longifolia (Micheli) Rudd, 1955 and Aeschynomene sp. prope villosa. They share certain similarities in size and architecture and showed a common characteristic: microsatellites distributed in metacentric (m), submetacentric (sm) and subtelocentric (st) chromosomes. The smallest chromosome pair constantly showed a displaced centromere (sm/st) in this group. *A. americana* and A. americana var. flabellata Rudd, 1955 carried satellites in the last pair (st), while in A. americana var. glandulosa (Poiret) Rudd, 1955 the position alternated between penultimate pair (sm) and the smallest pair (st) however shared the same karyotype: 8m + 1sm + 1st. These taxa showed slight variations in parameters such as THC, range and ratio. Although Aeschynomene sp. prope americana exhibited a karyotype formula 8m + 1sm + 1st the satellites were in pair six (m), while in Aeschynomene sp. prope villosa (7m + 2sm + 1st) were in pairs five and ten (Fig. 2). In addition, both taxa exhibited the highest THC in the series. On the other hand, A. villosa var. villosa showed two pairs of st chromosomes (4m + 4sm + 2st), consequently the most asymmetric karyotype of the group (CI = 34.07), but stood out for showing the greatest number of microsatellites (pairs 5, 6 and 10). In contrast A. villosa var. longifolia, without st chromosomes and with the largest number of sm chromosomes in the series (4m + 6sm) carried microsatellites in the smallest chromosomal pair (sm). In series Sensitivae, *Aeschynomene sensitiva* and *A. deamii* (Figs 1H, I, 2 and Table 2) shared relatively similar karyotypic formulae and exhibited only one pair of macrosatellites in the smallest chromosomal pair (sm) however in the latter the secondary constriction is so short that the associated satellite is almost imperceptible. The series Indicae, represented by *Aeschynomene evenia*, *A. scabra*, *A. rudis* and *A. ciliata* Vogel, 1838 showed three different chromosomal formulae in addition to one clear separation between karyotypes with one and two pairs of chromosomes with macrosatellites. The first two taxa: *A. scabra* and *A. evenia*, had a single SAT chromosomes pair, although in a different position, pairs 6 (m) and 10 (sm) respectively, while *A. rudis* and *A. ciliata* exhibited satellites in both pairs of smallest chromosomes (st and sm respectively) (Figs 1J–M, 2 and Table 2).

The species belonging to subgenus Ochopodium (Figs 1N–P, 2 and Table 2) showed, from the cytogenetic point of view, greater discrepancies. They presented well-differentiated karyotypic formulae and macrosatellites of variable aspect, position and number, in addition to having the highest values in THC and AC. These are species whose karyotypic asymmetry is related not only to the presence of displaced centromeres, but also to the greater differences in range and ratio (Table 2). This subgenus also includes *Aeschynomene paniculata*, the only taxon with satellites in the first pair, *A. amorphoides* Rose, 1894 with two pairs of satellites and *A. lyonnetii* whose macro-satellites are situated in the last pair, and for their volume and shape, makes it stands out from the rest of the taxa analyzed.

### Chromosomal variability and relationship patterns.

**Figure 3.**
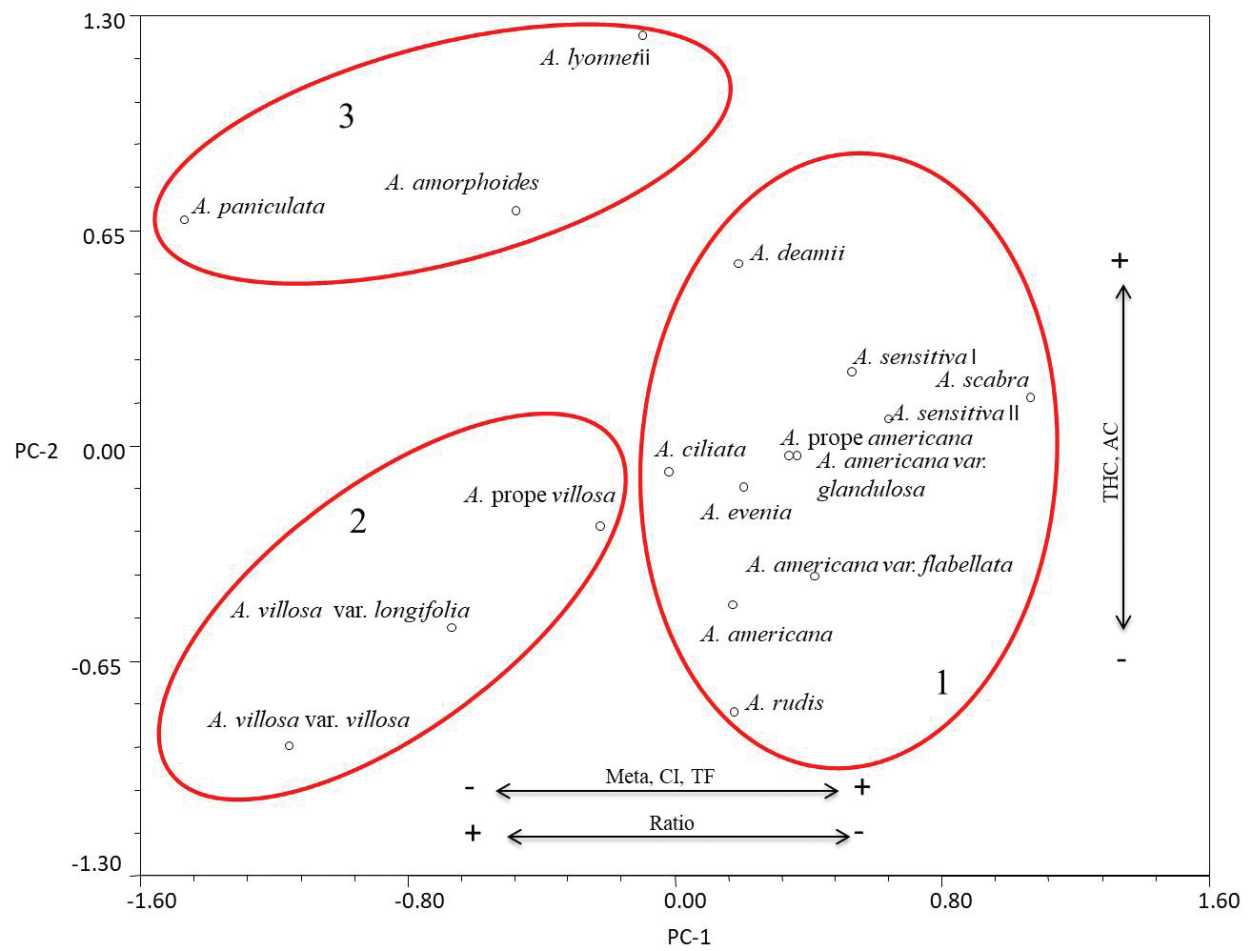
Projection of the 17 accessions of *Aeschynomene* onto the space of the first two principal components. Arrows indicate the patterns of variation in the characters with highest load. Abbreviations: AC = average chromosome size, CI = centromeric index, Meta = number of metacentric chromosomes, Ratio = major chromosome arm length/minor chromosome arm length, TF = index of asymmetry, THC = total haploid chromosomal length.

**Figure 4.**
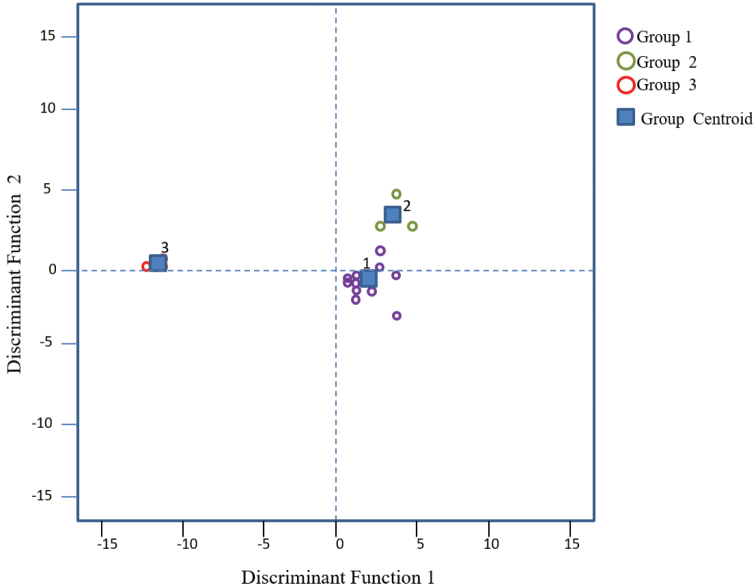
Groupings of the 17 accessions of *Aeschynomene* resulting from a Discriminant Function Analysis. Centroids indicate the average of the taxa in each group.

The graphic model (PCA) explains most of the variation in chromosomal characters. The characters with the highest load and determinants in the grouping pattern of the taxa were: the number of metacentric chromosomes (41.2935%) and THC (31.9768%). Together, these characters accumulated 73.2703% of the total variation. The PCA separated taxa under study into three groups (Fig. 3). Group 1 is made up of species from series Americanae (*A. americana*, its varieties and Aeschynomene sp. prope americana), Sensitivae and Indicae of subgenus Aeschynomene. This group is characterized by having a greater number of metacentric chromosomes, a higher centromeric index, higher values in asymmetric index and lower ratio values. Group 2 comprises A. villosa var. villosa, A. villosa var. longifolia and Aeschynomene sp. prope villosa of subgenus Aeschynomene; and is characterized by presenting lower centromeric indexes, lower values in TF, and higher ratio values. Group 3 includes species from series Pleuronerviae and Scopariae of *Ochopodium*, which are separated from the two previous groups, mainly because they have a greater total haploid chromosomal length (THC) and higher average chromosome sizes (AC), as well as higher ratio values. Discriminant function analysis (DFA) reinforces the preview showing that the groups identified by PCA are statistically significant (Tables 3, 4). The number of metacentric (m) and submetacentric (sm) chromosomes separates A. villosa var. villosa and relatives from the rest of the taxa included in the subgenus Aeschynomene while THC and AC separate the *Ochopodium* group from the previous two. The centroids of the three groups were clearly separated and there was no overlap between the species that constitute them (Fig. 4), which excluded classification errors in the analysis (Table 4).

**Table 3.** Results of the discriminant Function Analysis.

**Discriminant Function**	**Eigenvalues**	% **of Variance explained**	% **Cummulative**	**Canonical correlation**
1	36.501	92.7	92.7	0.987
2	2.887	7.3	100.0	0.862
**Derived Function**	**Wilks Lambda**	***Chi square***	**d.f.**	**Significance**
1 to 2	0.007	52.311	16	0.000
2	0.257	14.255	7	0.047

**Table 4.** Classification of the 17 accessions of *Aeschynomene* according to Discriminant Function Analysis.

Actual groups	Predicted groups							
	1		2		3		Total	
	Number	%	Number	%	Number	%	Number	%
1	11	100	0	0	0		11	100
2	0	0	3	100	0		3	100
3	0		0	0	3	100	3	100

### Identification of small isolated spherical structure and supernumerary NORs

**Figure 5.**
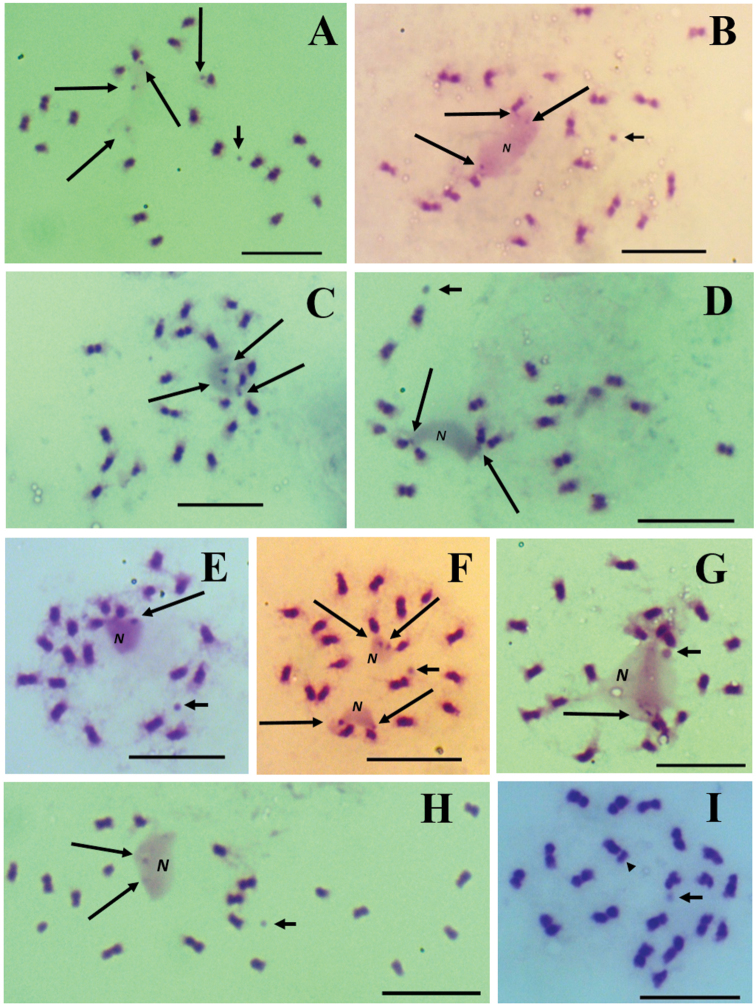
Chromosome rearrangements in Aeschynomene americana var. glandulosa (2n = 20). **A–H** Prometaphase. Chromosomal segments whose position suggests participation of the NOR function. The long arrows point to segments aligned or joined to the chromosomal arms by chromatin strands or embedded in one or two nucleoli (N) or in traces thereof. The short arrows highlight small isolated spherical structure. **I** Metaphase. The participation of the chromosomal segments decreases or ceases and only an isolated spherical structure is observed within the nucleus. The arrowhead points to a chromosomal fragment. Scale bars: 10 μm.

In Aeschynomene americana var. glandulosa the localization of the satellites in the karyotypes was often a difficult task as their position was alternated between the last two chromosomal pairs, sm and st respectively; representing a particular type of polymorphism that involves the secondary constriction and its satellite, although this transposition does not significantly alter the karyotype. In addition, nuclei in prometaphase and some metaphases frequently exhibited small isolated spherical structures with a density apparently different from that of the rest of the chromosomal complement. These structures of unknown nature were not found in the same position either associated or aligned with a particular chromosome and differ in size and shape from both the microsatellites described in series Americanae and the known chromosomal fragments (Fig. 5A–I). No similar structures were observed in any other taxon, even in A. villosa var. villosa where six satellites were found. Also, a complex sequence of rearrangements involving the presence of tiny chromosomal segments generally associated with one or two nucleoli or traces of these and apparently linked, without distinction of the arm, to chromosomes of different sizes by means of chromatin strands that were identified exclusively in nuclei in prometaphase (Fig. 5A–H).

## Discussion

The genera included in the tribe Dalbergieae share the same basic chromosome number x = 10, which presupposes a certain uniformity (Goldblatt 1981, Lavin et al. 2001, Mendonça Filho et al. 2002). However, our results showed that the species and infraspecific taxa of *Aeschynomene* possess uniform chromosome number and exhibit a wide karyotypic diversity (Fig. 1; Table 2). We found 10 karyotype formulae and great variation in the total haploid chromosomal lengths (from 11.39 μm in *A. rudis* to 22.41 μm in *A. amorphoides*), in the range (0.60 μm in *A. rudis* up to 1.82 μm in *A. paniculata*), the ratio (1.51 in *A. scabra* to 2.52 in *A. paniculata*), and CI (34.07 in A. villosa var. villosa to 43.11 in *A. sensitiva*). Furthermore, the number, size and position of secondary constrictions and satellites (SAT chromosomes) confirm the karyotypic heterogeneity in this group and its usefulness as markers for taxa even below the species level, particularly for those taxa difficult to define (Palomino and Vázquez 1991, Solís Neffa and Fernández 2002, Tapia-Pastrana and Tapia-Aguirre 2018). Its role in the organization of the nucleolus is obvious since secondary constrictions and satellites were often associated with projections of nucleolar material or even were observed immersed in a single nucleolus or in several small nucleoli, so here they are considered as nucleolar organizer regions (NORs). In this sense, secondary constrictions have been identified in different plant genera by in situ hybridization with rDNA probes and due to their correspondence with the SAT chromosomes it was possible to describe cytotypes in species and varieties with different levels of ploidy and even in hybrid taxa (Hasterok et al. 2001, Taketa et al. 2001, Kulak et al. 2002, Marasek et al. 2004, Hwang et al. 2011, Roa and Guerra 2012). In addition, the use of conventional cytogenetic techniques has proven its usefulness in the identification of SAT chromosomes for taxa discrimination that exhibit a high degree of intraspecific karyotype uniformity (Solís Neffa and Fernández 2002).

The above confirms the close association between major rDNA sites and SAT chromosomes (Pikaard 2000) and this agrees with the mapping of two 45S rDNA loci in the secondary constrictions of SAT chromosomes in *A. evenia*, particularly in the upper part of the AeLG10 linkage group (Chaintreuil et al. 2016b), that probably represents pair 10 in the karyotype of *A. evenia* obtained in our study (Figs 1K, 2).

The behaviour of the NORs in the form of secondary constrictions associated with satellites, as well as their size and position, has not been previously studied in species and infraspecific taxa in the genus *Aeschynomene*. Also, the location of the satellites, always in short arms, confirms a common tendency in the karyotypes of plant species where 86% of secondary constrictions are preferably located in short arms (Lima de Faria 1976, Lim et al. 2001) and particularly in Leguminosae (Biondo et al. 2006, Tapia-Pastrana 2012, Tapia-Pastrana and Tapia-Aguirre 2018 and literature therein cited).

Our results were in congruence with the classification based on morphological characters by Rudd (1955) for the New World species of the genus *Aeschynomene* and also with groupings based on phylogeny (Fig. 2). It is clear the presence of two groups that are separated by THC, AC, range and ratio; and whose entities correspond to the subgenera *Aeschynomene* and *Ochopodium*. It is likely that differences in THC, AC, and chromosome shape point to genomic differentiation processes through chromosomal evolution during speciation (Stebbins 1971, Kenton 1981, 1984, Grant 1989, Tapia-Pastrana et al. 2018). In our study, taxa having ACs about 2 μm, with the exception of *A. deamii*, series Sensitivae, belongs to the subgenus Ochopodium, which are perennial and occupy terrestrial habitat while those with ACs close to 1.5 µm belongs to the subgenus Aeschynomene and are annuals or short perennials and occupy semiaquatic habitats. Different investigations showed a close correlation between the life form, climatic and eco-geographic factors and genome size (Bennett 1972, 1976, Grime and Mowforth 1982, Ohri 1998, Bai et al. 2012 and literature therein cited). If considered that the THC expressed in μm is a good approximation to the size of the genome (Peruzzi et al. 2009, Harpke et al. 2015), then the subgenera *Aeschynomene* and *Ochopodium* could be another example in this regard.

In contrast, *A. deamii*, a perennial species, represents a particular case, because in spite of thriving in marshes and flooded areas and belonging to the group of species that nodulate in stem exhibits an exceptional THC (20.82 μm). Its chromosomal size, which corresponds to a high DNA content (1.93 pg) for a diploid species of the subgenus Aeschynomene (Arrighi et al. 2012) seems to correlate with large flowers (Rudd 1955) and with the height that exceeds 4 meters (Delgado-Salinas and Tapia-Pastrana, pers. obs.). *A. deamii* it was initially considered a tetraploid taxon, however, subsequent chromosomal counts corroborated a 2n = 20 (Arrighi et al. 2014). In addition to suggesting the existence of different chromosomal remodeling mechanisms involved in the evolution of its karyotype, our observations support its location as a monospecific lineage in an ITS-based phylogeny (Chaintreuil et al. 2018). Similar karyotype characteristics with *A. sensitiva*, the blackening of the stems and on drying fruits, as well as a calyx with whole or almost whole lips, justify so far, its location in the series Sensitivae (Fig. 2).

The karyotype analysis demonstrated being helpful in the infrageneric delimitation and exhibited a close association not only with the previous morphological and taxonomic groupings, but with phylogenetic trees obtained with molecular markers. Our results suggest the possibility of adding new taxonomic categories, particularly in the series Indicae, since it can clearly be separated into two subseries with species that exhibit one (*A. scabra* and *A. evenia*) and two (*A. rudis* and *A. ciliata*) pairs of chromosomes with satellites. This idea is corroborated by the complements of *A. denticulata* Rudd, 1955 (series Indicae) that also exhibit a pair of SAT chromosomes (data not shown).

**Figure 6.**
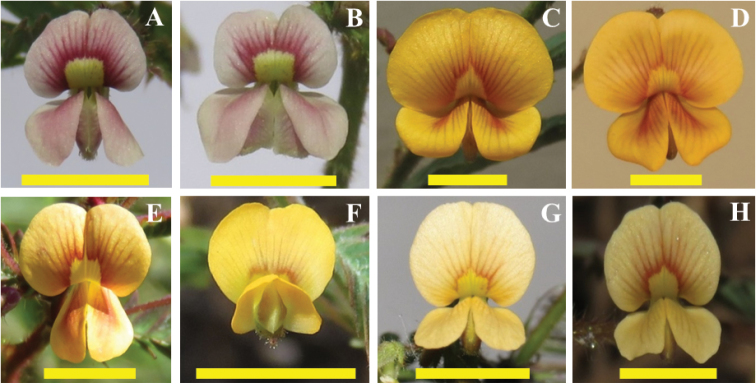
Floral morphotypes of taxa of the series Americanae. **A, B***Aeschynomene americana***C**A. americana var. flabellata**D**A. americana var. glandulosa**E**Aeschynomene sp. prope americana**F**A. villosa var. villosa**G**A. villosa var. longifolia**H**Aeschynomene sp. prope villosa. Scale bars: 5 mm.

In series Americanae taxa are morphologically related and difficult to identify, however the karyotypes of the species and infraspecific taxa show their own identity (Fig. 2) in accord to the Nod-dependent American clade recovered by Chaintreuil et al. (2013). Apparently, we detected a group of morphologically related taxa where the karyotypic differences observed between the species and their varieties in this series are consistent with the idea that we are dealing with a set of non-described taxa that require being review taxonomically. PCA on karyotype characteristics and the morphological differences observed in herbarium specimens, in the descriptions of habits and ways of life and discrepancies in both the floral morphotypes and the geometry of the maculae on the banner petal support this proposal (Fig. 6). In this sense, a more accurate evaluation of these floral morphotypes would provide valuable information for future taxonomic revisions.

The different location of NOR also suggests that A. americana var. glandulosa undergoes chromosomal remodeling *via* breaks in regions close to secondary constrictions and subsequent transposition of the nucleolus organizer regions; as well as the participation of tiny chromosomal segments whose location inside the nucleolus would indicate not only an active contribution of the NOR function, but also a dynamic state of chromatin remodeling. Such segments could be described as satellites except for the fact that they are not observed in metaphase nuclei or in corresponding stages in nuclei of closely related taxa. On the other hand, the presence of small isolated spherical structures of unknown nature, separated from both the nucleolus and chromosomes, frequently observed in the nuclear space of metaphase cells resembles extrachromosomal circular DNA (eccDNA) detected by electron microscopy in plants, and whose size ranges from 0.1 μm to more than 5 μm in contour length with an average of 1.7 μm for *Triticum aestivum* Linnaeus, 1753 and 1.5 μm for *Nicotiana tabacum* G.Don, 1838 respectively (Kinoshita et al. 1985) and containing sequences derived mainly from repetitive chromosomal DNA (Cohen et al. 2008). The contribution of eccDNA to the evolution and plasticity of plant genomes is unclear and, although there is currently no direct experimental evidence, it is speculated that it is involved in the evolution of B chromosomes and in the mobility of rDNA (Cohen et al. 2008). They also resembles the satellite-like structures recorded in chromosomes of prometaphase cells stained with Giemsa of *Nicotiana kawakamii* Y. Ohashi, 1976 (Nakamura et al. 2001) or well to the minichromosomes observed by fluorescent in situ hybridization (FISH) in metaphase chromosomes of interspecific marsupial hybrids (Metcalfe et al. 2007 in Fig. 5B). It is known that inter- or intraspecific hybridization events lead to genomic instability, which results in *de novo* chromosomal rearrangements due to changes in chromatin structure among other aspects (Fontdevila 1992, 2005, Metcalfe et al. 2007). Thus, our evidence could indicate that A. americana var. glandulosa is actually a homoploid hybrid (Nieto Feliner et al. 2017). However, a more accurate interpretation of the nature and function of such structures will have to wait for the application of molecular cytogenetic methods.

Moreover, variations in the number and position of NORs (supernumerary NORs) without some other major karyotypic changes have been reported in *Allium cepa* Linnaeus, 1753 (Sato 1981), *A. fistulosum* Linnaeus, 1753 and its hybrids (Schubert et al. 1983, Schubert 1984, Schubert and Wobus 1985, Pich et al. 1996). Likewise, supernumerary NORs, all in subterminal position, have been found in *Allium flavum* Linnaeus, 1753 (Loidl and Greilhuber 1983) and in natural populations of *A. schoenoprasun* Linnaeus, 1753 (Bougourd and Parker 1976). In addition, *Turnera sidoides* Linnaeus, 1767, exhibits a high degree of intraspecific karyotype uniformity and the subspecies are distinguished only by the number, type and position of the satellites (Arbo 1985, Solís Neffa and Fernández 2002).

Thus, the genome plasticity exhibited in the nuclei of A. americana var. glandulosa, including the possible participation of supernumerary NORs, would explain the variability in karyotype morphology shown by a group of taxa identified as *A. americana*. It would also support the taxonomic proposal to recognize so-called *Aeschynomene americana* complex; however, this must also be confirmed with molecular cytogenetic studies in a greater number of populations and species.

In series Sensitivae, *Aeschynomene deamii* and *A. sensitiva* exhibit relatively similar karyotypes with macrosatellites in the last pair (Fig. 1H, I and Table 2). In addition, between the two accessions of *A. sensitiva*, slight differences are observed in parameters such as THC, AC, range and ratio; however, they does not substantially affect neither karyotype nor CI, this suggests the loss of genetic interaction between these two populations or, adaptations to different eco-geographic factors.

Within series Indicae, both *A. ciliata* and *A. rudis* are easily identified by the presence of macrosatellites in both pairs of smallest chromosomes (Figs 1L, M, 2). Likewise, similarity in their CIs indicates a close relationship. The main difference was found in the chromosomal size, since *A. rudis* showed the lowest THC (11.39 μm) in our investigation (Fig. 1 and Table 2), which may be reflect to the eco-geographic characteristics of place of collection (the Salta Province, Argentina, the Southern Hemisphere). In this respect it is worth mentioning that although this species has been described with really large flowers, the Argentinian collections reviewed by Rudd (1955) had exhibited smaller flowers than those from latitudes farther north. The small size of their chromosomes should encourage population studies throughout their distribution to support a proposal that at that time lacked solid arguments about the inclusion of infraspecific categories in this taxon (Rudd 1955). Differences in flower sizes associated with changes in DNA contents were observed in the African *A. schimperi* (Chaintreuil et al. 2016a), while Verdcourt (1971) suggested that specimens of *Aeschynomene* with large flowers could be of polyploid origin. On the other hand, *A. scabra*, which exhibits the most symmetrical karyotype (TF = 45.54) within taxa under study differs from *A. evenia* (species of difficult morphological identification), not only by exhibiting larger chromosomes, but by the position of satellites in pair 6 (m) and not in pair 10 (sm), respectively (Fig. 2). It should be noted that in the Nod-independent nodulation clade (Chaintreuil et al. 2013) *Aeschynomene deamii* and *A. sensitiva* (series Sensitivae) appear as sister species of *A. ciliata*, *A. scabra* and *A. rudis* (series Indicae). Our results show that these five species, together with *A. evenia*, besides being associated by other cytogenetic parameters (Figs 1, 2; Table 2), share the characteristic of exhibiting macrosatellites in the short arms of generally small chromosomes, which clearly differentiates them from series Americanae (Nod-dependent American clade recovered by Chaintreuil et al. 2013) that exclusively exhibits microsatellites. In this sense, there is a concordance with the proposal derived from molecular studies.

*A. paniculata* (series Pleuronerviae) is the only species that exhibits macrosatellites in the short arms of the first chromosomal pair as well as the largest number of submetacentric chromosomes (seven), so it represents a distinctive case not only within subgenus Ochopodium, but throughout the genus *Aeschynomene*.

Series Scopariae includes *A. amorphoides* and *A. lyonnetii*, which bear little resemblance, judging from their different karyotype formulae, CI, and the number and shape of their satellites (Fig. 1O, P, Table 2).

It is pertinent to point out that the scarce chromosomal homology exhibited between the species of the two previous series seems to correspond to the polytomy observed in the Ochopodium clade in the phylogeny by Chaintreuil et al. (2013) and suggests the need for a new taxonomic revision.

Comparatively, our results show that the morphology and particularly the chromosomal size of the species included in the subgenus Ochopodium are more similar to those recorded in *Dalbergia spinosa* Roxburgh, 1814 (Jena et al. 2004) than to those of the subgenus Aeschynomene. However, the meaning of these types of comparisons should await the detailed karyotypic description in a greater number of species included in *Aeschynomene* and *Dalbergia* Linnaeus f., 1782. On the other hand, the fact that the higher THC, AC and ratio (Table 2) are found in the taxa included in *Ochopodium* indicate that it is a different group, which responds to different adaptations derived from its forms of life and/or the type of environment in which they are developed (Petrov 2001, Chaintreuil et al 2016a). Current phylogenies place *Ochopodium* close to *Machaerium* Persoon, 1807 and *Dalbergia* and propose their phylogenetic separation. However, the scarce reliable karyotypic information in these last two and the limited sampling in our study do not allow to support this proposal at this time from the cytogenetic perspective.

In this way, we show that karyotype information is useful in the taxonomic delimitation of the genus and its value can be extended to other genera of Dalbergieae*sensu lato* as research on chromosomal structure progresses.

## Conclusions

The predominantly diploid species of the New World and the lack of an aneuploidy compared to the tetraploid and octoploid African species seem to confirm the New World origin of *Aeschynomene*. Although polyploidy has played an important role in the evolution of the genus, our results indicate that speciation in *Aeschynomene* has also been accompanied by chromosomal remodeling events, as well as subtle changes in the number and position of secondary constrictions and associated satellites, and that these changes preceded duplications and aneuploidies previously recorded in species distributed in the New and Old World. Therefore, the karyotype comparison is a reliable way in identification and classification in *Aeschynomene* since it generally agrees with the morphological series and even with the recent relationship hypotheses that indicate that *Ochopodium* should separate from *Aeschynomene* and constitute a new genus, although the latter must be corroborated by studies that include a greater number of species.

In addition, the identification of isolated small spherical structures and the finding of a complex sequence of rearrangements that could involve supernumerary NORs support the proposal that these elements model the chromosomal evolution of this subgroup in an unsuspected manner. *Aeschynomene* exhibits in both subgenera a high diversity of karyotypes that allow observing patterns of chromosomal evolution associated to important events in the divergence of lineages that have been detected in previous molecular studies. Such is the case of the species of the series Indicae which are grouped within Nod-independent clade and have been also proposed as parental taxa of allopolyploids, although attempts at hybridization have failed to form fertile individuals.
